# National Dental Association

**Published:** 1898-11

**Authors:** 


### Reports of Society Meetings.

### NATIONAL DENTAL ASSOCIATION.

The First Annual Meeting of the National Dental Association was held in Omaha, Nebraska, August 30 to September 2, 1898.

The meetings were held in the Creighton Medical Building, where ample facilities were afforded for sessions, section meetings, exhibits, clinics, etc. With the President, Dr. Thomas Fillebrown, occupying the chair, the meeting was called to order at 11 A.M., Tuesday, August 30, and opened with prayer by Professor J. Taft, of Cincinnati.

The mayor of Omaha, Hon. Frank N. Moore, in welcoming the delegates, alluded in a humorous manner to the many addresses he had been obliged to make, and then welcomed the dental convention to Omaha, and trusted the members would enjoy their stay in the city and also the Trans-Mississippi Exhibition. He presented the symbol of the freedom of the city in two massive gilded wooden keys.

These were accepted by the President with expression of thanks, who then called upon Dr. James Truman to respond to the mayor.

Dr. Truman said he voiced the feeling of the audience in thanking the mayor and, through him, the citizens of Omaha for their hospitable reception. He then briefly alluded to the progress made in dentistry in the past fifty years and the union of the two wings, North and South, the final culmination of which was being perfected at this meeting, and now we recognize no North, no South, no East, and no West, but united we aim to accomplish the work given us to do. He called attention to the fact that the State of Nebraska a few short years ago was the home of the wildest of the aboriginal tribes. These to-day were assembled here at Omaha in a Congress of Peace, one of the most interesting features of this great Exposition which had made Omaha famous throughout the length and breadth of the land.

In closing he said we cannot longer locate the West. It is not to be found at Chicago, neither at Omaha, nor at Denver, Salt Lake, Tacoma, Seattle, Portland, or San Francisco. It has passed out of the Golden Gate and centred upon the Hawaiian group; not there, but facing the Orient, the flag of our country waves over the Philippines, the emblem of freedom, of intelligence, and of the development of humanity, and neither one power nor an aggregation of powers can lower it, for the West of this country is there to remain for the enlightenment of the nations.

The usual routine business was then taken up.

In offering the report of the Recording Secretary, Dr. George H. Cushing, the President spoke in feeling terms of the absence of this long-honored official. Dr. C. N. Peirce offered a resolution tendering a telegraph message of regrets to Dr. Cushing. The resolution was adopted and Dr. Peirce appointed a "committee of one" to carry it into effect.

The Committee on Credentials reported that certain States had ignored Section 4 of Article IV. in the election of delegates to this Association. On motion of Dr. S. W. Foster, it was resolved that the rule be strictly enforced.

On motion of Dr. G. V. I. Brown, the privileges of the floor were tendered to all delegates present who were ruled out from membership by the enforcement of this rule.

The Secretary read a communication from Dr. R. Ottolengui on the subject of amendments to the patent laws, asking the endorsement of the National Dental Association for this movement. The communication was referred to the Executive Committee, which subsequently reported unanimously the following resolution:

"WHEREAS, The Supreme Court having already declared all such patents as are contemplated in this move to amend the patent law invalid, which makes all such amendments unnecessary; and

"WHEREAS, The constant agitation of this question is detrimental to the best interests of organizations in existence; therefore be it

"*Resolved*, That the National Dental Association now in session disapproves of any further work in this direction, and recommends that the whole question be dropped as unwise and unnecessary."

The resolution was presented to the Association and unanimously adopted by that body.

Dr. B. Holly Smith, Vice-President from the South, taking the chair, the President then read his annual address, of which we give a brief abstract. Dwelling upon the value of organization as essential to the success of any movement in which the combined efforts of several individuals are desired, he said, "It converts the irresponsible mob into the disciplined and responsible army. It is the fly-wheel of progress, which gives balance to combined effort, brings up and pushes forward the laggard of conservatism, restrains the erratic flight of the exuberant enthusiasm of the reformer, gives steadiness to action, and makes advancement sure. It holds the special privileges of the individual subject to the will of the body for the good of the whole. Nations, peoples, and communities require formulated rules for their government and control."

He then traced the evolution of law, from the statute law, changeable by the will of the body which made it, to organic or constitutional law, requiring a more complicated proceeding to change it, common laws being those deduced from generally accepted principles, and not the result of legislative action.

Laws are the measure of progress. Good laws are not found in a corrupt age, while a cultivated and enlightened people will not tolerate evil laws. Applying these principles to our own professional organization, it will be found that our organizations of the past were as good as the material of which they were formed would afford. Our present Association will be no better than the culture and enthusiastic efforts of its members will make it. Constitutions will not furnish brains, culture, nor application; standing rules will not compel research nor write papers. Neither, on the other hand, can by-laws prevent the student from seeking out the hidden mysteries of nature. The things most essential to the success of our organization are brains, education, application, enthusiasm, and an unselfish devotion that will bring to and lay on the altar of science the best efforts of every member. "All that I have I give unto thee" is the motto of the truly professional spirit, and must be the governing principle of a progressive scientific body.

Professional relations must be governed by an entirely unselfish principle, and to live where life is controlled by it, regulated by good common sense, then no loss will come.

The President spoke of the two distinctly new features of our present constitution,—the creation of the divisions of the East, the West, and the South, a feature of cosmopolitan character, which must awake wider interest in the meetings, guarding against sectional control. The provision for the formation of branches is the second distinctively new feature, through the influence of which it may reasonably be expected that large numbers will become actively and permanently connected with the Association, working in harmony with it, who would not otherwise join its ranks.

It has made this National Association possible, bringing into harmonious action and sympathy the sentiment and energies of a reunited profession. Its practical and successful working has been shown by the Southern branch during the present year, and repetitions of such success may be prophesied with branches working in harmony with and as parts of the parent body, forwarding their proceedings for publication in the annual volume of the Association; a complete record representing the scientific progress of the profession during the year will thus be furnished, to be placed on the shelves of the library of every member.

He spoke of the manner of securing delegates as perhaps too complicated, the refusal of delegates from other than State societies probably keeping many good men out of our ranks.

The true value of professional organization lies in the wider diffusion of knowledge, culture, and refinement, and a true professional spirit. Hence, open wide the doors and go out into the streets and by-ways and compel men to come to the feast. Giving is gaining, teaching is learning,—no one can get from another the full benefit of his thought and teaching until he has grasped the hand and felt the personality of the leader and teacher. Honor is accorded to those who impart their knowledge the most freely.

Of the future of the Association he said, "To me it appears especially bright and encouraging. Whatever of shadows may have hung over our profession in the past, whatever of evil may have attended on its way, whatever of prejudice, ambition, or adverse interests may still exist, there is light upon its pathway in the future. The passing through the desert must needs have been made, but now on the heights a radiant pathway lies before us. 'The spirit of the age is with it, and it is one with the potent forces that advance knowledge, shape thought, and mould the life of nations.' The future promises much for the increase of scientific knowledge, the advancement of professional interests, and the promotion of the welfare of humanity."

The Committee on National Museum and Library reported that having visited the institution in the interests of the Association, they had been cordially received, finding the officials interested in the work, and expressing a willingness to do their utmost to build up the dental sections. A highly creditable amount of work in accumulating specimens has been accomplished, while the library contains the best and most complete collection of dental literature in the world. But few men in the profession appreciate this great work in elevating dentistry in the estimation of scientific men.

The recommendation of Surgeon-General George M. Sternberg, endorsed by the Secretary of War, for the employment of a dental pathologist, passed the Senate, but failed in committee of conference. The recommendation will, however, be renewed and submitted to Congress at its next session.

The committee appointed to take into consideration the establishment of a journal devoted to the interests of the Association reported that they had given the matter consideration, and believed that, if properly carried out, it would be of great value, both to the profession in general and to the Association, as a vehicle through which papers and proceedings of the various State and branch associations would reach the members of the dental profession throughout the civilized world. The income from subscriptions and advertisements would probably not be sufficient to meet expenses for the first few years, and the deficiency would devolve upon the Association. The committee, however, has in view a scheme by which they think the work can be established and carried out, but this is not yet wrought out in all its details. The committee, therefore, asked to be continued another year, which was granted.

(To be continued.)

